# BIN1 Is Decreased in Sporadic but Not Familial Alzheimer’s Disease or in Aging

**DOI:** 10.1371/journal.pone.0078806

**Published:** 2013-10-21

**Authors:** Elizabeth B. C. Glennon, Isobel J. Whitehouse, J. Scott Miners, Patrick G. Kehoe, Seth Love, Katherine A. B. Kellett, Nigel M. Hooper

**Affiliations:** 1 School of Molecular and Cellular Biology, Faculty of Biological Sciences, University of Leeds, Leeds, United Kingdom; 2 Dementia Research Group, School of Clinical Sciences, Institute of Clinical Neurosciences, University of Bristol, Bristol, United Kingdom; Nathan Kline Institute and New York University School of Medicine, United States of America

## Abstract

Bridging integrator 1 (BIN1) has been implicated in sporadic Alzheimer’s disease (AD) by a number of genome wide association studies (GWAS) in a variety of populations. Here we measured BIN1 in frontal cortex samples from 24 sporadic AD and 24 age-matched non-dementia brains and correlated the expression of this protein with markers of AD. BIN1 was reduced by 87% (p=0.007) in sporadic AD compared to non-dementia controls, but BIN1 in sporadic AD did not correlate with soluble Aβ (r_s_=-0.084, p=0.698), insoluble Aβ (r_s_=0.237, p=0.269), Aβ plaque load (r_s_=0.063, p=0.771) or phospho-tau load (r_s_=-0.160, p=0.489). In contrast to our findings in sporadic AD, BIN1 was unchanged in the hippocampus from 6 cases of familial AD compared to 6 age-matched controls (p=0.488). BIN1 declined with age in a cohort of non-dementia control cases between 25 and 88 years but the correlation was not significant (r_s_=-0.449, p=0.081). Although BIN1 is known to have a role in endocytosis, and the processing of the amyloid precursor protein (APP) to form amyloid-β (Aβ) peptides is dependent on endocytosis, knockdown of BIN1 by targeted siRNA or the overexpression of BIN1 in a human neuroblastoma cell line (SH-SY5Y) had no effect on APP processing. These data suggest that the alteration in BIN1 is involved in the pathogenesis of sporadic, but not familial AD and is not a consequence of AD neurodegeneration or the ageing process, a finding in keeping with the numerous GWAS that implicate BIN1 in sporadic AD. However, the mechanism of its contribution remains to be established.

## Introduction

Genome-wide association studies (GWAS) have recently identified a number of genes that are related to sporadic AD [1-8]. These genes have implicated several molecular and cellular processes in the development of AD, including immune system functions, lipid metabolism and processes at the cell membrane such as endocytosis [4]. Bridging integrator 1 (BIN1) maps to the long arm of human chromosome 2 (2q14) and encodes multiple tissue-specific isoforms of the Myc-interacting adapter protein [9,10]. BIN1 has been implicated in sporadic AD by a number of GWAS in a variety of populations [2,3,5-7,11,12]. BIN1 was originally implicated in sporadic AD by the association with the disease of two single nucleotide polymorphisms (SNPs), rs744373 and rs7561528, both of which lie approximately 30 kb upstream of the BIN1 gene [3,6], a finding subsequently replicated in a number of different cohorts [2,7,11]. In addition, a novel 3 bp insertion allele 28 kb upstream of BIN1 (rs59335482) was also recently identified [12]. There are several alternatively spliced variants of BIN1, the different isoforms being expressed by different tissues [10]. The widely expressed isoform of BIN1 is located in the nucleus and is thought to have functions relating to cell cycle regulation and cancer, whereas the brain-specific isoform is located in the axons and at the nodes of Ranvier [13]. One of the numerous roles of BIN1 is in endocytosis [14-16].

Despite the identification of BIN1 gene variants as being associated with sporadic AD, little is known about BIN1 protein expression and its involvement in AD pathogenesis. In this study we measured BIN1 protein level in frontal neocortex from cases of sporadic AD and age-matched control brain samples, the hippocampus of familial AD cases compared to age-matched controls, and in a non-dementia cohort across a range of ages. We demonstrate that BIN1 protein level is significantly decreased in sporadic AD compared to age-matched controls, but not in familial AD. Although BIN1 level tended to decline with age, this trend was not significant. As BIN1 has a function in endocytosis, we investigated its potential role in amyloid precursor protein (APP) metabolism and amyloid-β (Aβ) generation in a cell culture system. Neither knocking down endogenous BIN1 expression with targeted siRNA nor overexpression of BIN1 in a human neuroblastoma cell line had an effect on APP processing.

## Results

### BIN1 is decreased in sporadic but not familial AD and does not correlate with age

**Figure 1:**
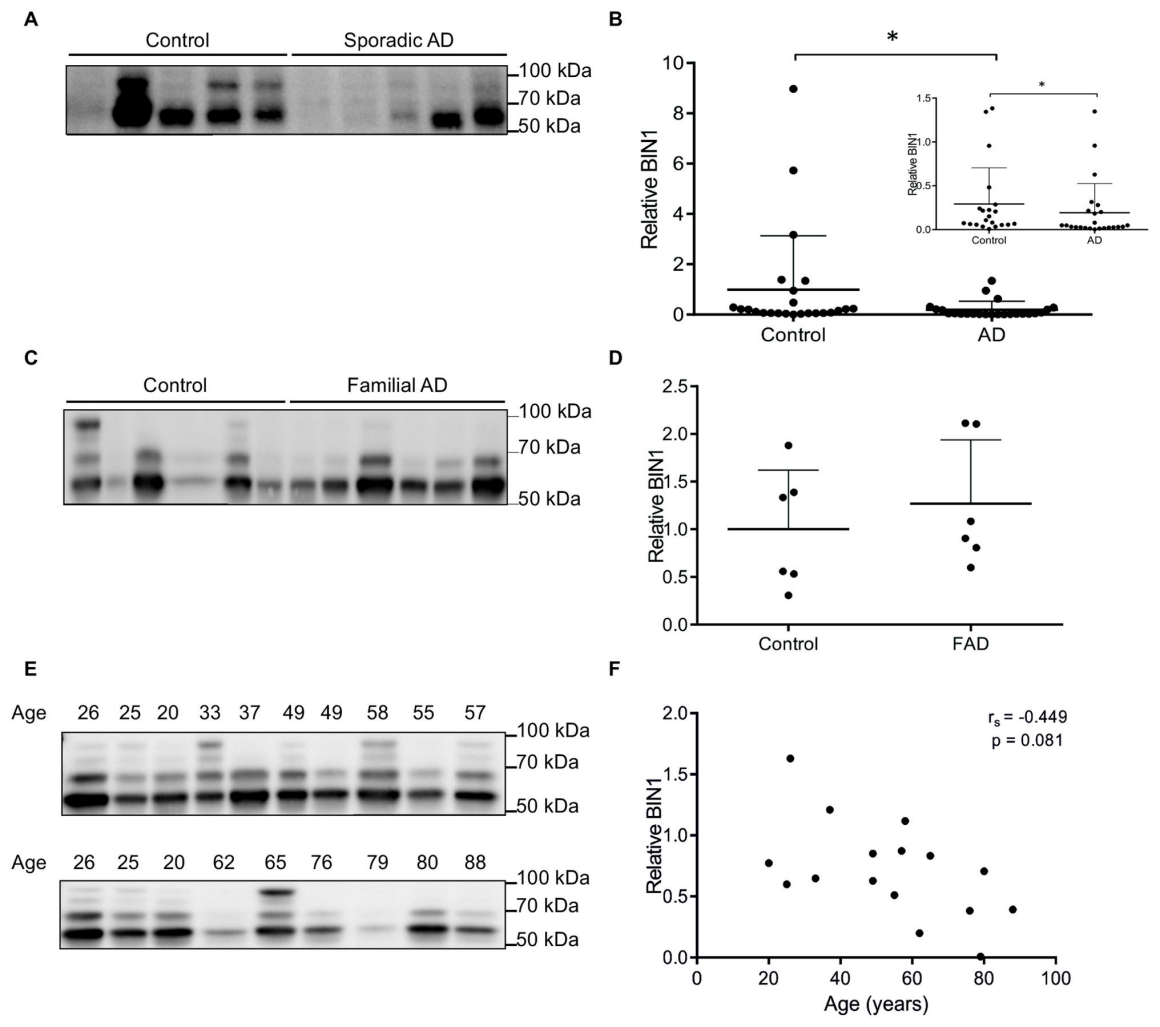
BIN1 is decreased in sporadic AD, but not in familial AD and does not correlate with age. Representative immunoblot of BIN1 in temporal cortex samples from sporadic AD patients compared to age-matched controls (A) with densitometric analysis of all bands relative to total protein represented in a group scatter plot, n = 24 per group, Mann Whitney U (B) and data omitting the 3 outlying control samples n = 21 control, n = 24 AD, Mann Whitney U (inset B). Line represents mean, error bar represents SD, *p<0.05. Immunoblot of BIN1 in hippocampal samples from familial AD patients compared to age-matched controls (C) with densitometric analysis relative to total protein represented in a group scatter plot (D). Line represents mean, error bar represents SD, n = 6 per group, Indepentent T-test. Immunoblot of BIN1 in hippocampal samples of a series of non-dementia brains covering a wide spectrum of ages (E) with densitometric analysis relative to total protein, n = 16 (F). BIN1 does not correlate with age as determined by Spearman’s rank correlation coefficient (r_s_).

Quantitative immunoblotting was used to assess BIN1 level in the frontal cortex in sporadic AD and in age-matched non-dementia controls (Table 1). BIN1 appears as multiple bands on immunoblot, with the bands corresponding to BIN1 spliced isoforms, the largest of which is 95kDa [9,10,17,18]. BIN1 was significantly reduced in sporadic AD by a mean of 87% compared to controls (mean ± SD; controls 1.00 ± 2.13 and AD samples 0.19 ± 0.33, p = 0.007) (Figure 1A and B). To ensure that 3 outlying control samples were not causing disproportionate data bias, the cohort was reanalysed omitting these samples. BIN1 remained significantly reduced in sporadic AD, by a mean of 34% (mean ± SD; controls 0.29 ± 0.41 and AD samples 0.19 ± 0.33, p = 0.031) (Figure 1B inset). There was no significant difference in age between the sporadic AD and control cases (mean age ± SEM; 82.5 ± 1.4 years and 76.5 ± 2.7 years, respectively, p = 0.204) and the omission of the 3 outlying control samples did not significantly change the age of the control group (76.3 ± 3.1 years). In addition, there was no significant difference in the level of neuron-specific enolase (NSE) between the sporadic AD and control samples (mean ± SEM; 0.51 ± 0.05 pg/mL for each group, p = 0.992), indicating that the reduction in BIN1 in the sporadic AD samples was not caused by neuronal loss. The post-mortem delay was also not significantly different between the sporadic AD and control group (mean ± SEM; 51.08 ± 4.11 h and 38.50 ± 6.27 h, respectively, p = 0.100).

**Table 1:** Characteristics of the sporadic AD and control cases used in the study.

**Gender**	**Age (y)**		**APOE ε4 allele**	**Braak stage**	**PM delay (h)**
**Sporadic AD**					
M	69		4.4	5	48
F	70		3.4	6	25
M	74		3.4	5	50
F	74		4.4	5	53
F	77		3.4	4	43
F	78		4.4	6	77
F	78		3.4	5	9
F	78		3.3	6	35
M	79		3.4	6	28
M	80		3.4	6	31
F	81		3.4	6	42
F	81		3.3	4	66
F	83		3.4	5	43
M	85		3.4	4	58
M	85		3.4	6	66
F	87		3.4	5	72
F	87		2.4	5	67
F	88		3.3	6	79
F	89		3.3	5	71
F	89		3.3	5	82
F	90		4.4	4	21
F	91		3.4	4	37
F	91		2.4	5	70
F	96		2.3	4	53
**Control**					
F	43		-	-	12
F	48		2.4	-	79
M	53		3.3	-	7
M	62		3.4	0	4
M	64		3.3	2	23
F	72		3.3	0	24
M	73		3.3	-	36
M	77		2.3	3	10
M	78		3.3	2	12
M	79		3.3	-	24
M	80		3.3	3	106
F	80		3.3	0	92
F	81		3.3	2	103
M	82		3.3	2	30
M	82		3.3	2	3
F	82		4.4	2	37
M	82		3.3	2	56
M	84		3.3	3	48
F	84		2.3	1	17
F	88		3.3	2	62
F	88		3.3	2	28
M	90		2.3	2	45
M	90		3.3	-	48
F	93		3.3	2	18

To determine whether the reduction in BIN1 expression is a primary disease process or secondary to other AD-associated changes, we measured the level of BIN1 in brain tissue in familial AD and age-matched controls (Table 2). In hippocampus from cases of familial AD there was a slight (27%) increase in BIN1 compared to the age-matched controls but this did not reach statistical significance (mean ± SD; controls 1.00 ± 0.62 and familial AD samples 1.27 ± 0.67, p = 0.488) (Figure 1C and D). There was no significant difference in age between the familial AD and control cases (mean age ± SEM; 61.8 ± 4.3 years and 63.2 ± 6.4 years, respectively, p = 0.866). The post-mortem delay was significantly different between the familial AD and control group (mean ± SEM; 13.3 ± 3.28 h and 40.7 ± 8.67 h, respectively, p = 0.015). We showed previously that there is no significant difference in the expression of the APP between the familial AD and control group [19] and there was also no significant difference in the expression of the neuronal marker MAP2 (p = 0.188), indicating that the reduction in BIN1 is not caused by neuronal loss.

**Table 2:** Characteristics of the Familial AD and control cases used in the study.

**Gender**	**Age (y)**		**Cause of death**	**PM delay (h)**	**Mutation**
**Familial AD**					
M	61		Bronchopneumonia	15	APP717(Val-Gly)
F	69		Bronchopneumonia	10	APP717(Val-Ile)
F	62		Bronchopneumonia	23	APP717(Val-Ile)
M	42		Bronchopneumonia	6	PS1 DELTA4
F	72		Unknown	4	APP717(Val-Ile)
F	65		Pneumonia	22	PS1 (E280G)
**Control**					
F	62		Haemothorax	81	-
M	57		Left ventricular failure	45	-
F	79		Chronic obstructive airway disease	38	-
M	37		Acute necrotic pancreatitis	27	-
M	79		Septicaemia secondary to faecal peritonitis	24	-
M	65		Coronary artery occlusion	29	-

Mutation refers to the familial AD mutation identified in these individuals.

We also used quantitative immunoblotting to measure the level of BIN1 in a series of non-dementia brains covering a wide spectrum of ages (Table 3). The level of BIN1 in the hippocampus tended to decline with age but the correlation was not significant (Figure 1E and F, r_s_=-0.449, p = 0.081). We showed previously that there is no significant correlation between age and the expression of APP [19] and there was no significant correlation between age and the expression of MAP2 (r_s_=0.322, p = 0.223).

**Table 3:** Characteristics of the series of non-dementia brains covering a wide spectrum of ages used in the study.

**Gender**	**Age (y)**	**Cause of death**	**PM delay (h)**
F	26	Carcinoma of the lung	44
M	25	Ruptured aortic arch aneurysm	18
F	20	Sudden accident	38
F	33	Pulmonary embolus	96
M	37	Acute necrotic pancreatitis	27
M	49	Pulmonary oedema	44
M	49	Coronary artery occlusion	32
M	58	Myocardial infarction	23
M	55	Syringomyelia	24
M	57	Left ventricular failure	45
F	62	Haemothorax	81
M	65	Coronary artery occlusion	29
F	76	Congestive heart failure	28
M	79	Septicaemia secondary to faecal peritonitis	24
F	80	Left ventricular failure/ bronchopneumonia	31
F	88	Carcinomatosis	43

### BIN1 does not correlate with soluble or insoluble Aβ, Aβ plaque load or phospho-tau load in sporadic AD samples

**Figure 2:**
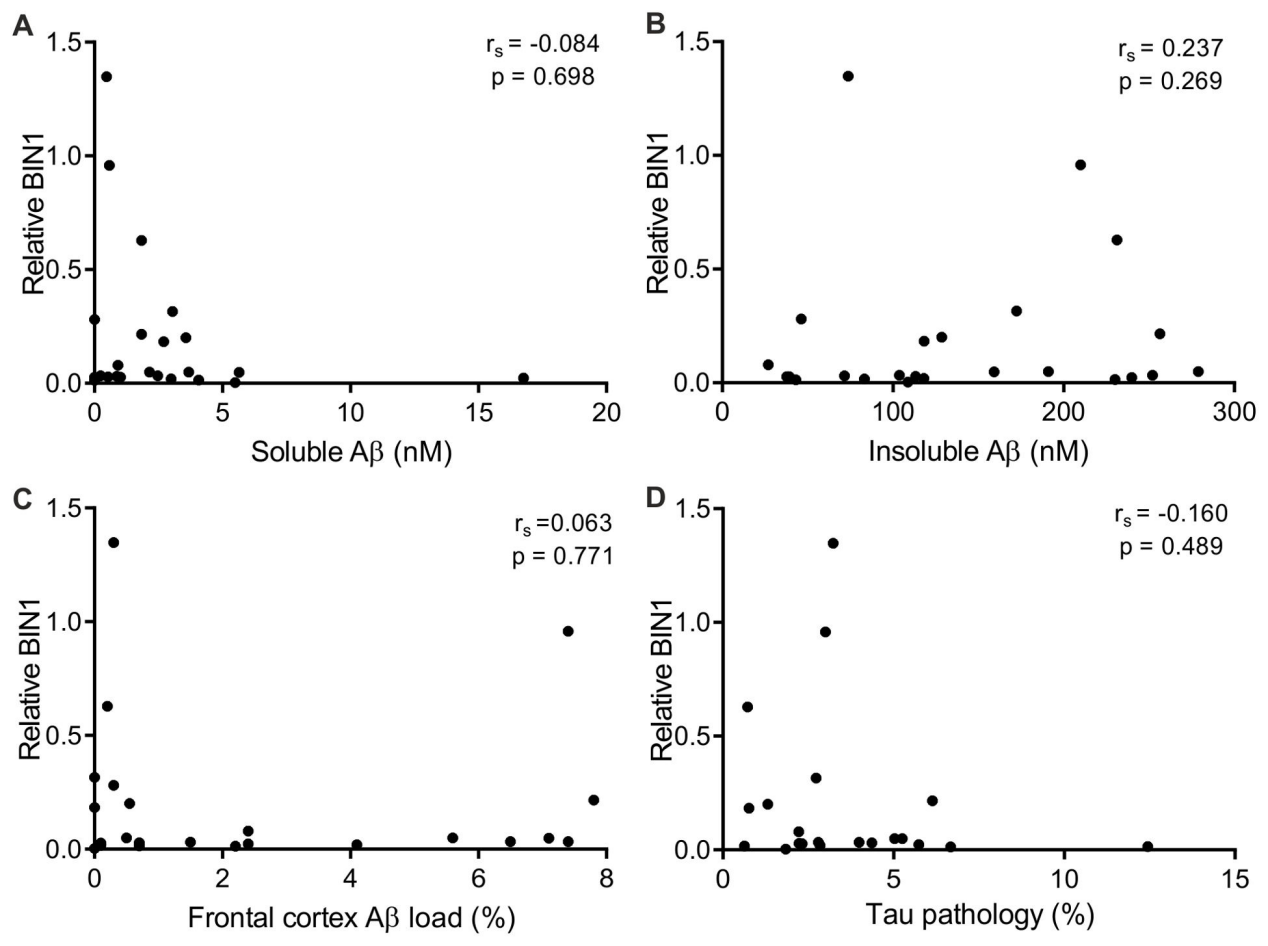
BIN1 does not correlate with soluble or insoluble Aβ, Aβ load or Tau pathology in sporadic AD samples. Relative BIN1 protein levels of sporadic AD samples were plotted against soluble Aβ (A), insoluble Aβ (B), Aβ load (C) and tau pathology (D) for each subject in the cohort (n = 24). BIN1 does not correlate with soluble Aβ, insoluble Aβ, Aβ load and tau pathology as determined by Spearman’s rank correlation coefficient (r_s_).

As BIN1 is decreased in sporadic AD compared to age-matched controls (Figure 1A and B) we investigated whether there was a correlation between BIN1 and the levels of soluble and insoluble Aβ and the Aβ plaque and phospho-tau load in the sporadic AD cases. Neither soluble nor insoluble Aβ correlated with BIN1 (Figure 2A and B, r_s_=-0.084, p=0.698 and r_s_=0.237, p = 0.269, respectively). In addition, Aβ plaque load, as measured immunohistochemically, did not correlate with BIN1 (r_s_=0.063, p = 0.771, Figure 2C). Recently, Chapuis et al. (2013) proposed that BIN1 mediates AD risk by modulating tau pathology [12]. However, in our cohort the phospho-tau load, as measured immunohistochemically, did not correlate with BIN1 protein level (r_s_=-0.160, p = 0.489, Figure 2D). In addition BIN1 protein levels did not correlate with post-mortem delay (r_s_=0.373, p = 0.072).

### BIN1 overexpression has no effect on APP processing

**Figure 3:**
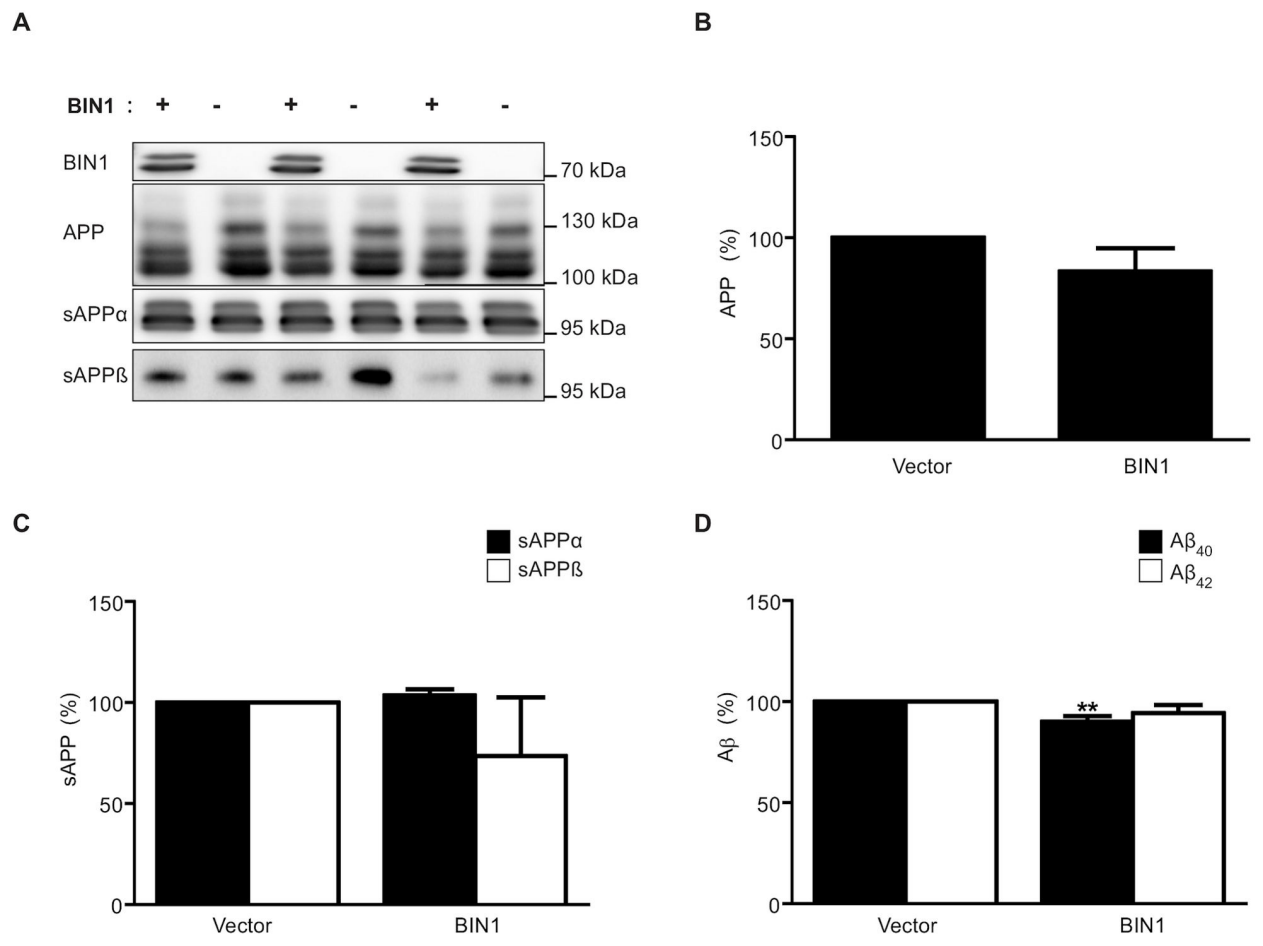
Effect of BIN1 overexpression on APP processing. SH-SY5Y cells were transfected with either BIN1 cDNA (+) or empty vector (-). Representative immunoblots of cell lysates (BIN1 and APP) and conditioned media samples (sAPPα and sAPPβ) (A). Densitometric analysis of APP (B) and sAPPα and sAPPβ (C) relative to total protein. ELISA analysis of Aβ40 and Aβ42 in conditioned media (D). Data represent mean ± S.D., n=3, *p<0.05, Independent T-test.

As BIN1 is known to play a role in endocytosis [14-16] and the amyloidogenic processing of APP requires the endocytosis of APP [20-23], to investigate whether BIN1 affects the processing of APP the neuronal isoform of BIN1 was overexpressed in SH-SY5Y cells (Figure 3A). BIN1 overexpression did not alter the level of APP in the cell lysate (Figure 3A and B) nor the production of the soluble ectodomain resulting from α (sAPPα) or β (sAPPβ) cleavage (Figure 3A and C) in conditioned media, compared to empty vector control. BIN1 overexpression caused a small but significant 10% decrease in Aβ40 peptide in the conditioned media compared to the empty vector control (mean ± SD; control 100% and BIN1 over-expression 90.60% ± 2.68, p = 0.005); Aβ42 was also decreased, by 6% (mean ± SD; control 100% and BIN1 over-expression 94.45% ± 3.96), but this did not reach significance (Figure 3D).

### BIN1 knockdown has no effect on APP processing

**Figure 4:**
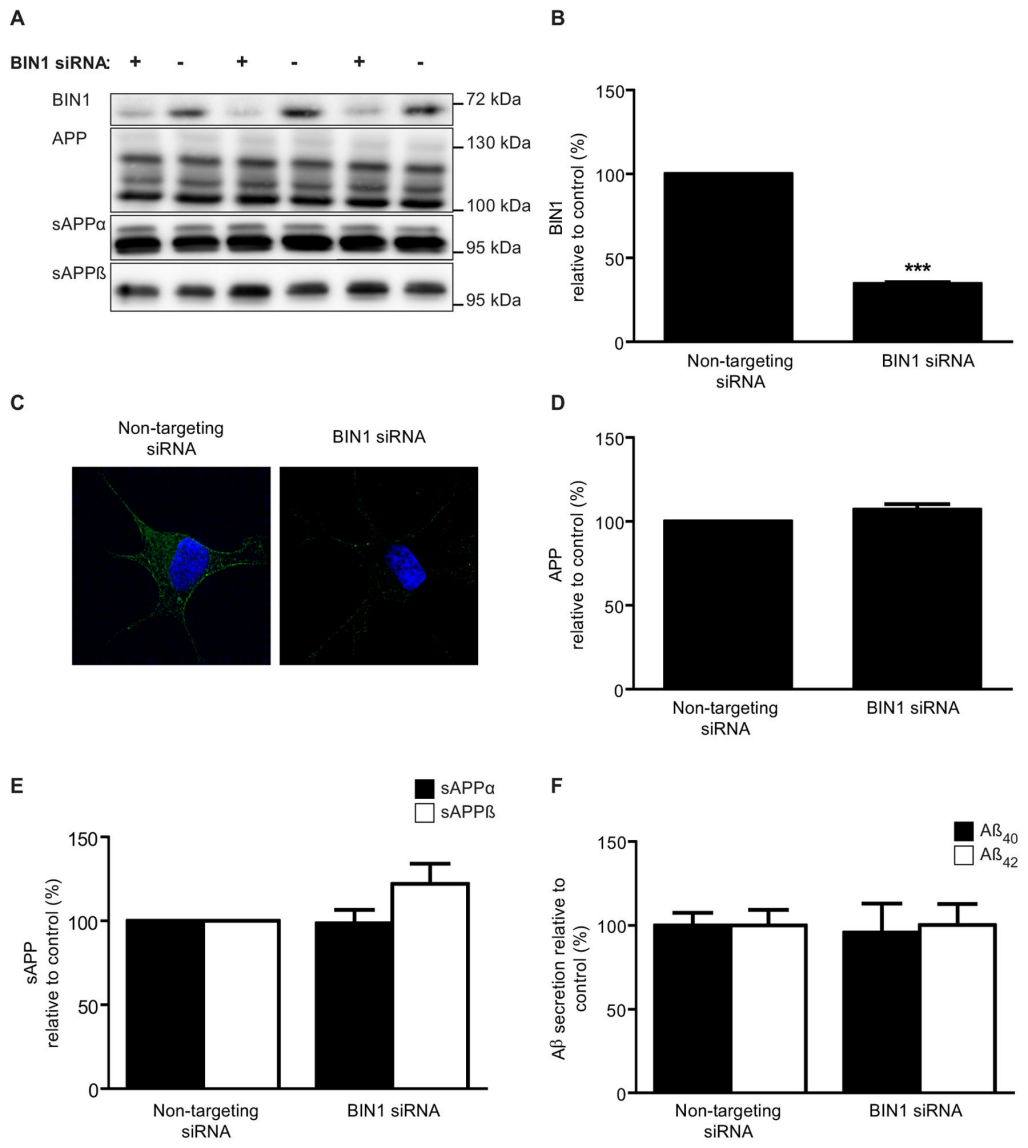
BIN1 knockdown has no effect on APP processing. SH-SY5Y cells were transfected with either siRNA against BIN1 (+) or non-targeting control siRNA (-). Representative immunoblots of cell lysates (BIN1 and APP) and conditioned media samples (sAPPα and sAPPβ) (A). Fluorescence microscopy images of BIN1 (green) and DAPI staining (blue) (C). Densitometric analysis of BIN1 (B), APP (D) and sAPPα and sAPPβ (E) relative to total protein. ELISA analysis of Aβ40 and Aβ42 in conditioned media (F). Data represent mean ± S.D., n=3, *p<0.05, Independent T-test.

To confirm the results of the BIN1 overexpression on APP processing the converse experiment was performed in which endogenous BIN1 expression was knocked down by siRNA in the SH-SY5Y cells. BIN1 knockdown caused a 65% (p < 0.001) reduction in BIN1 protein when SH-SY5Y cells were incubated with BIN1 siRNA compared to a non-targeting siRNA control (Figure 4A and B). Fluorescence microscopy revealed that BIN1 was localised to the plasma membrane and cytoplasm and this staining was virtually abolished when BIN1 expression was knocked down by siRNA (Figure 4C). BIN1 knockdown had no significant effect on APP expression in the cell lysate (Figure 4A and D); on the production of sAPPα or sAPPβ (Figure 4A and E); or on the level of Aβ peptides (Figure 4F) in the conditioned media.

## Discussion

In this study we have shown that the level of BIN1 protein is lower in the brain in sporadic AD than in non-demented age-matched controls. There was a large variation of BIN1 protein levels across the whole cohort of sporadic AD and control samples, however, this is unsurprising as protein levels are heritable molecular phenotypes that exhibit considerable variation between individuals, populations and sexes [24]. The observation that BIN1 protein is reduced in sporadic AD brains builds on recent observations from numerous GWAS where variants in the BIN1 gene were found to be associated with sporadic AD [2,3,5-7,11,12]. As the identified SNPs lie ~30kb upstream of the start of BIN1 [3,6,12], a change in expression level is unsurprising, as the primary and three dimensional structure of BIN1 would not be expected to be altered. A recent study showed that in a large cohort (64 AD and 61 control cases), BIN1 mRNA level was higher in AD cases than age-matched controls [12]. Although this was a robust study with an impressive cohort size, it was not specified whether the AD cases had sporadic or familial disease. Indeed we have shown that in contrast to the finding in sporadic AD, BIN1 protein expression remains unchanged in familial AD compared to age-matched controls. Notably, whereas Chapius et al. (2013) measured mRNA [12], we measured the level of BIN1 protein, which is likely to be more biologically and clinically meaningful. Indeed, several studies have shown that levels of mRNA transcript do not necessarily equate to protein levels [25,26].

The levels of BIN1 protein were reduced in the frontal cortex of sporadic AD compared to age-matched non-dementia controls, but not in the hippocampus of familial AD. This implies that the alteration in BIN1 is involved in the pathogenesis of sporadic, but not familial AD and is not a consequence of AD neurodegeneration. The brain areas analysed did differ between the sporadic and familial AD cohorts, but both the frontal cortex and the hippocampus are areas known to be associated with AD [27-29]. Each cohort had its corresponding age-matched non dementia controls, however, we can not preclude the possibility that the difference between sporadic and familial AD in BIN1 levels is due to the different brain areas investigated. The familial AD cases used in this study all have either a mutation in APP (APP717 Val-Gly or Val-Ile) or presenilin 1 (PS1 DELTA4 or E280G). However, there are numerous mutations in APP, presenilin 1 and presenilin 2 which can cause familial AD [30] and our data do not preclude a change in the level of BIN1 in familial AD caused by other mutations. Ageing is the greatest risk factor for AD [31] but our data indicate that BIN1 expression does not change with age; suggesting that the changes in BIN1 associated with sporadic AD are not a result of ageing. 

Our data show that BIN1 does not correlate with the amount of tau pathology in sporadic AD. This is in contrast to a recent publication that proposed that a 3 bp insertion allele 28 kb upstream of BIN1 (rs59335482) was associated with the tau load in AD brains [12]. Over half (53.7 %) of the cohort in that study had at least one copy of the rs59335482 insertion allele and the insertion was associated with tau load but not with Aβ40 or Aβ42 in the brains of AD patients. Chapius et al. showed that *Amph*, the BIN1 *Drosophila* ortholog, modulates Tau neurotoxicity *in vivo* in a *Drosophila* model system, the loss of *Amph* was able to suppress Tau-induced neurotoxicity [12]. In addition, they showed by co-immunoprecipitation techniques that BIN1 interacts with Tau in synaptosomal fractions from mouse brain homogenates, however they did not detect any colocalisation of BIN1 with neurofibrillary tangles by immunohistochemical analysis in the brain of AD cases, suggesting that BIN1 probably does not have a role in the late stages of tangle formation although it could affect tau aggregation and/or oligomer formation in earlier stages of disease [12]. This may explain why we did not detect a correlation between BIN1 and tau in sporadic AD brain.

One of the numerous roles of BIN1 is in endocytosis [14-16] . Endocytosis has an important role in AD pathology and is involved in Aβ generation [22], Aβ toxicity to neurons [32], and Aβ clearance from the brain by transport of Aβ across the blood brain barrier [33]. We have shown that the BIN1 level in sporadic AD does not correlate with soluble Aβ, insoluble Aβ or Aβ load in the temporal cortex, implying that decreased BIN1 is unlikely to affect endocytosis-dependent aspects of the generation or degradation of Aβ. To corroborate this, we have shown using cell line models, that BIN1 knockdown or overexpression does not alter APP processing, although there is a small but significant decrease in secreted Aβ40 when BIN1 is overexpressed in SH-SY5Y cells. Although BIN1 did not significantly and consistently affect the generation or degradation of Aβ in our experiments, compensatory mechanisms for the reduced amount of BIN1 in sporadic AD could be at play. During endocytosis BIN1 forms a heterodimer with amphiphysin 1, a BIN1 homologue [16]. Amphiphysin 1 is capable of forming homodimers in the absence of BIN1 [16], so amphiphysin 1 could compensate for decreased BIN1 in sporadic AD and upon siRNA-mediated depletion of BIN1.

In conclusion our data demonstrate that BIN1 is reduced in sporadic but not familial AD, showing that the alteration in BIN1 is probably a primary contributor to the disease and not a secondary consequence of AD. Our findings are consistent with the numerous GWAS that implicate variation in the gene encoding for BIN1 in sporadic AD. Finally, we did not find evidence that a decline in BIN1 is likely to contribute to the increasing risk of AD with age.

## Materials and Methods

### Ethics statement

Brain tissue was obtained from the HTA licensed South West Dementia Brain Bank, University of Bristol, UK (sporadic AD) and from the Medical Research Council London Neurodegenerative Diseases Brain Bank, Institute of Psychiatry, King’s College London, UK (familial AD and age-spectrum control cohort). The study was conducted with approval from The Cardiff Research Ethics Committee, North Somerset and South Bristol Research Ethics Committee and the Leeds Central Research Ethics Committee. 

### Study cohorts

All sporadic AD cases had been subjected to detailed neuropathological examination. Sporadic AD cases had been assessed according to the criteria of the Consortium to Establish a Registry for Alzheimer's Disease (CERAD) [34]. The sporadic controls had no history of cognitive decline or dementia, showing the absence of AD (as defined by CERAD) or other neuropathological abnormalities. The levels of total soluble and total guanidine-extractable Aβ [35,36], the Aβ plaque load (area fraction of cerebral cortex immunopositive for Aβ) [37,38], NSE level [39] and phospho-tau load (area fraction of cerebral cortex immunopositive for phospho-tau) [37,40] had previously been measured in all cases. The AD and control groups were matched as far as possible for post-mortem delay, age-at-death and gender, as presented in Tables 1, 2 and 3.

### Tissue preparation

For measurements of BIN1 protein and NSE in sporadic AD samples, approximately 200 mg of frontal neocortex (Brodmann area 6) was homogenised in 1 ml lysis buffer (0.5% Triton X-100, 20 mM Tris/HCl pH 7.4, 10% (w/v) sucrose containing aprotinin (1 µg/ml) and phenylmethane sulfonyl fluoride (PMSF; 10 µM)) (all reagents from Sigma-Aldrich, Pool, Dorset, UK). Brain tissue was homogenised for 30 s in a Precellys 24 automated tissue homogeniser (Stretton Scientific, Derbyshire, UK) with 2.3-mm silica beads (Biospec, Thistle Scientific, Glasgow, UK) and total protein was measured using Total Protein kit (Sigma-Aldrich, Pool, Dorset, UK). The homogenates were centrifuged at 20 817 *g* for 15 min at 4°C, and aliquots of the supernatant were stored at -80°C until used. Familial AD and age-spectrum control brain tissue samples from the hippocampus were homogenised in 9 volumes of phosphate buffered saline (PBS without Ca^2+^ and Mg^2+^, Gibco), 0.5% (v/v) Nonidet P40, 0.5% (w/v) sodium deoxycholate, pH 7.4 in the presence of a complete protease inhibitor cocktail (Roche Applied Science, Burgess Hill, UK) using an electrical homogeniser. Samples were centrifuged at 14,000 *g* for 10 min. 

For measurements of Aβ, tissue (200 mg) was allowed to thaw to 4°C, homogenised in 5 volumes (w/v) of Tris-buffered saline (TBS) extraction buffer [140 mM NaCl, 3 mM KCl, 25 mM Tris/HCl, pH 7.4, containing 1% Nonidet P-40 (NP40), 5 mM EDTA, 2 mM 1,10-phenanthroline, 10 µM PMSF and 1 µg/ml aprotinin (all reagents from Sigma-Aldrich, Pool, Dorset, UK), as detailed in [35,36]. The homogenate was then centrifuged at 20 817 *g* for 15 min at 4°C and the supernatant (soluble fraction) was stored at −80°C until used. The pellet was homogenised in 6.25 M guanidine HCl in 50 mM Tris/HCl, pH 8.0, incubated for 4 h at 25°C and centrifuged at 20 817 *g* for 20 min at 4°C. The resultant supernatant (guanidine-extractable fraction) was stored at −80°C until used.

### Immunoblotting

The protein content of the brain homogenate was determined using bicinchoninic acid in a microtitre plate-based assay with bovine serum albumin (BSA) standards. Samples were mixed with an equal volume of SDS dissociation buffer (125 mM Tris/HCl, pH 6.8, 2% (w/v) SDS, 20% (v/v) glycerol, 100 mM dithiothreitol, 0.002% (w/v) bromophenol blue), and boiled for 4 min. Proteins were resolved by SDS polyacrylamide gel electrophoresis using 7% (APP) and 10% (BIN1, sAPPα and sAPPβ) polyacrylamide gels. Resolved proteins were transferred to Immobilon P polyvinylidene difluoride membrane (Amersham, Little Chalfont, UK). The membrane was blocked by incubation for 1 h with PBS containing 0.1% (v/v) Tween-20 and 5% (w/v) dried milk powder. Antibody incubations were performed in PBS Tween containing 2% (v/v) bovine serum albumin. Antibody against BIN1 (Abcam, Cambridge, UK) was used at 1:1000, anti-APP antibody 22C11 (MercMillipore, Billerica, MA, USA) was used at 1:2500, anti-sAPPα antibody 6E10 (Covance, New Jersey, USA) was used at 1:4000 and 1A9 antibody (gift from Dr I Hussain, GlaxoSmithKline, Harlow, UK) was raised against a neoepitope formed on wild type sAPPβ following β-cleavage of APP was used at 1:2500. Horseradish peroxidase-conjugated secondary antibody was used at 1:4000 in the same buffer. Bound antibody was detected using the enhanced chemiluminescence detection method (Amersham Biosciences, Amersham, UK). Densitometric analysis was performed using Image J 1.44p (National Institutes of Health, USA). Quantification was in relation to total protein. Total amount of protein was determined from amido black (0.1% (w/v) amido black, 1% (v/v) acetic acid) stained membranes using Image J 1.44p.

### Measurement of total soluble and insoluble (guanidine-extractable) Aβ

The method of ELISA measurement of total soluble and insoluble Aβ was reported previously [35,36]. Soluble and insoluble (guanidine-HCl-extractable) fractions were analysed by sandwich ELISA in which monoclonal anti-Aβ (4G8 clone, raised against amino acids 18-22; Millipore, Watford, UK) was used for the capture step and biotinylated anti-human Aβ monoclonal antibody (10H3 clone) (Thermo Fisher Scientific, Northumberland, UK) for the detection step.

### Measurement of Aβ load

Parenchymal Aβ load had previously been measured in all cases [38]. The field fraction (percentage area occupied by Aβ) was measured in an unbiased selection of 10 areas of cortex covering 4mm^2^ with the help of Histometrix software (Kinetic Imaging, Wirral,UK) driving a Leica DM microscope with a motorised stage. Aβ-laden blood vessels were excluded from analysis.

### Measurement of tau load

The precentage area of cerebral cortex immunopositive for phospho-tau had also been measured previously [37,40], using the same unbiased selection and image analysis method as that used to measure Aβ load (see above).

### Cell culture

SH-SY5Y (human neuroblastoma) cells were cultured in Dulbecco’s Modified Eagle Medium (DMEM) (Lonza, Basel, Switzerland) supplemented with 10% (v/v) fetal bovine serum (FBS) (Biosera, East Sussex, UK), in a humidified incubator at 37°C with 5% (v/v) CO_2_. 

### Plasmid and siRNA transfection

cDNA encoding human BIN1 in expression vector pcDNA3.1(+) (Source Bioscience, Nottingham, UK) or empty vector alone (Invitrogen life sciences, Paisley, Scotland, UK) was introduced by electroporation into SH-SY5Y cells stably expressing APP_695_ in pIREShyg [41]. Cells were selected using 1 mg/mL G418 (Sigma-Aldrich, Pool, Dorset, UK).

siRNA specific for human BIN1 and a non-targeting sequence (UGGUUUACAUGUCGACUAA UGGUUUACAUGUUGUGUGA UGGUUUACAUGUUUUCUGA UGGUUUACAUGUUUUCCUA) were obtained as smartpools from Dharmacon (Thermo Fisher Scientific Biosciences, Northumberland, UK). SH-SY5Y cells were seeded in routine culture medium and allowed to adhere overnight. Cells were transfected with 50 nM (final concentration) BIN1 or non-targeting control siRNA as a complex with Dharmafect 3 (Thermo Fisher Scientific Biosciences, Northumberland, UK) according to the manufacturer’s instructions and incubated for 24 h at 37°C with 5% (v/v) CO_2_. 

### Cell preparation

Cells were incubated for 48 h in OptiMEM reduced sera medium (Invitrogen life sciences, Paisley, Scotland, UK) at 37°C with 5% CO_2_ (v/v). OptiMEM reduced sera medium was removed from the cells and centrifuged for 10 min at either 3913 *g* at 4°C. The supernatant was either stored at -20°C for Aβ ELISA or concentrated ~50 fold using Vivaspin 100,000 Da molecular weight cut off polyethersulfone membrane concentrator (Sartoris Stedim Biotech, Surrey, UK) by centrifugation at 3913 *g* at 4°C. 

Cells were washed and harvested in PBS (with Ca^2+^ and Mg^2+^), and pelleted by centrifugation at 3193 *g* at 4°C. Cells were lysed in RIPA lysis buffer (50 mM Tris, 150 mM NaCl, 0.5% (w/v) Sodium deoxycholate, 1% (v/v) NP-40, pH 8.0) with EDTA complete protease inhibitor cocktail (Roche Diagnostics, West Sussex, UK), incubated at 4°C for 30 min and centrifuged at 13,000 *g* for 10 min at room temperature. The subsequent supernatant was frozen at -20°C for protein analysis. 

### Aβ ELISA

Sandwich ELISAs for the detection of human Aβ40 and Aβ42 were performed as described previously [42]. Briefly, 96-well microtitre plates were coated overnight at 4°C with primary antibodies against Aβ40 (33.1.1) and Aβ42 (2.1.3.35.86) (a kind gift from C. and E. Eckman* and P. Das, Mayo Clinic, Jacksonville, FL, USA; *current address Atlantic Health System, Morristown, NJ, USA). Following blocking and incubation with conditioned media, bound Aβ peptides were detected with HRP-conjugated detection antibody (Aβ40, 13.1.1-HRP (C. and E. Eckman and P. Das); Aβ42, 4G8-HRP (Covance, New Jersey, USA)). 

### Fluorescence Microscopy

Fluorescence microscopy was carried out as described previously [43]. Briefly, cells were cultured in normal growth medium to ^~^60% confluence on glass coverslips and siRNA treated (see above). Post-incubation, cells were fixed with 4% (v/v) paraformaldehyde for 10 min, permeabilised using 0.2% (v/v) Triton X-100 and blocked overnight at 4 °C in PBS containing Ca^2+^ and Mg^2+^ ions with 5% (v/v) fish skin gelatin. Coverslips were then incubated for 2 h in the same buffer containing BIN1 specific antibody (Abcam, Cambridge, UK), washed, and then incubated with the appropriate fluorescent probe-conjugated secondary antibody under the same conditions. Nuclei were counterstained by washing briefly in DAPI stain, and coverslips were mounted onto glass slides using Fluoromount-G (Southern Biotech). Cells were visualised using a DeltaVision Optical Restoration Microscopy System (Applied Precision). 

### Statistical analysis

The distribution of the AD cases compared to their age-matched controls was determined by the Kolmogorov-Smirnov test. Group data were compared using either an Independent T-test for parametric, or a Mann-Whitney U test for non-parametric data 2-tailed Spearman’s rank correlation coefficient was used on non-parametric data to assess the correlation between BIN1 and soluble and insoluble Aβ, Aβ load, tau load and age; p ≤ 0.05 was considered significant. The data were analysed using the Statistical Package for Social Sciences (SPSS 17.0) program (Chicago, USA) and GraphPad Prism (version 6) (Graphpad Software Inc , California, USA).
